# Establishing Barriers to and Enablers of Nurse-Enabled Subcutaneous Therapy Self-Administration Programs for Patients With Myeloma: Protocol for a Qualitative Descriptive Study

**DOI:** 10.2196/85053

**Published:** 2025-12-23

**Authors:** Hayley Beer, Lisa Guccione, Amit Khot, Simon Harrison, Meinir Krishnasamy

**Affiliations:** 1 Department of Haematology, Peter MacCallum Cancer Centre and Royal Melbourne Hospital, 305 Grattan St, Melbourne, Victoria, 3000, Australia, +61 3 85595079; 2 Department of Advocacy, Myeloma Australia, Burnley, Australia; 3 Centre for Health Services Research and Implementation Science, Peter MacCallum Cancer Centre, Melbourne, Australia; 4 Sir Peter MacCallum Department of Oncology, University of Melbourne, Melbourne, Australia; 5 Department of Nursing, School of Health Sciences, Faculty of Medicine, Dentistry and Health Sciences, Melbourne University, Melbourne, Australia; 6 Victorian Comprehensive Cancer Centre Alliance, Melbourne, Australia

**Keywords:** multiple myeloma, self-administration, nurse-enabled therapy, subcutaneous therapy, implementation science

## Abstract

**Background:**

Multiple myeloma (MM) is associated with the greatest symptom burden of all hematological cancers and, despite substantial improvements in treatment options with high response and survival rates, is still considered incurable, with patients undergoing multiple lines of therapy over many years. Subcutaneous (SC) injections are a common mode of delivery for current and future MM therapy, with evidence suggesting that programs that give patients or carers responsibility for administration can bring benefits to the patient and health care system by reducing the number of required visits to hospital.

**Objective:**

This study will explore and describe barriers to and enablers of implementing nurse-enabled SC therapy self-administration programs for patients with MM to develop a road map for national scalability.

**Methods:**

This qualitative descriptive study is informed by the Consolidated Framework for Implementation Research. Participants included key stakeholders from across Australia, including patients, carers, health professionals, and policymakers with experience of implementation, facilitation, and participation in nurse-enabled SC therapy self-administration programs. Data were collected via virtual focus groups or semistructured interviews and analyzed using the framework method to identify barriers and enablers. The Expert Recommendations for Implementing Change matching tool will be used to develop strategies to target barriers and enhance enablers, informing the development of a national road map.

**Results:**

This study was funded in March 2024 and approved by Peter MacCallum Cancer Centre Human Research Ethics Committee in May 2024. Data collection was conducted between June 2024 and November 2024. A total of 32 participants were recruited. Data analysis is underway, with results expected to be published in February 2026.

**Conclusions:**

To our knowledge, this study will be the first of its kind to identify and compare barriers to and enablers of implementing nurse-enabled SC self-administration programs for patients with MM. Applying the Consolidated Framework for Implementation Research to guide study processes provides an evidence-informed approach to understanding how discrete and intersecting factors influence program implementation and sustainability, informing the development of a comprehensive implementation road map. As the availability of SC therapies grows for other cancers and chronic diseases, this model of care could serve as a blueprint for broader applications, impacting patient quality of life and optimization of health care use.

## Introduction

### Background

Multiple myeloma (MM) is the second most prevalent hematological cancer worldwide. It is largely a disease of older adults, with a mean age at diagnosis of 70 years [1]. The 5-year relative survival rate for MM has increased from 26% in the period between 1988 and 1992 to 56% in the period between 2014 and 2018 [2]. Despite these advances, MM remains an incurable disease characterized by prolonged, continuous periods of treatment targeting its relapsing, remitting nature. However, improved disease control has brought new challenges, including extended periods of specialist hospital surveillance and increased burden of travel, inconvenience, and cost for patients and carers, especially for those from regional, rural, and remote areas [3].

The emotional, physical, and financial impact of living with MM is considerable for both the patient and those caring for them. Patients report a complex combination of disease- and treatment-related side effects that impact health outcomes, including decreased physical functioning (98.9%), cognitive impairment (80.2%), and financial difficulties (78.4%) [4]. The consequences of MM also place a considerable burden on carers, who fear for the future and experience disruption to routine, decreased ability to work due to time spent in hospital, and worries about end of life [5,6].

Having the ability “to care for myself” is recognized as one of the most prevalent patient-reported unmet needs among people with incurable cancer [7,8]. Information and knowledge to help manage the impact of cancer and its treatment has been proven to ameliorate anxiety and improve quality of life and motivation to self-care. This may be especially beneficial for people living with MM and their caregivers [9,10].

The evidence summarized above points to a need to develop contemporary models of care responsive to the long-term challenges faced by people affected by MM. One opportunity to address the impact of MM on the lives of patients and carers is to reduce the burden of frequent travel to specialist centers for treatment. The option for self-administration of subcutaneous (SC) injectable therapy offers an opportunity to decrease the impact of treatment burden and its time toxicity [11], with additional favorable impacts on health care resource use.

### Bortezomib Self-Administration Program

Bortezomib is a proteasome inhibitor used in the treatment of newly diagnosed and relapsed MM. It is delivered via SC injection over approximately 20 seconds [12]. Our team implemented and sustained a nurse-enabled subcutaneous therapy self-administration program (NEST-SP) for patients receiving bortezomib, wherein patients or a nominated carer are offered the opportunity to learn how to administer their SC bortezomib injection at home. This program has resulted in avoidance of 3 out of 4 weekly hospital visits for patients and carers per treatment cycle. Initial evaluation data from the first 20 patients showed that attending the hospital for their injection resulted in an average time commitment of 4.5 hours per treatment day compared with 6 minutes when self-administering the injection at home [13]. These findings also demonstrated an opportunity for considerable savings for patients regarding costs of transport, parking, and food incurred when visiting the hospital [13]. Further evaluation of 70 patients in the program revealed that self-administration saved approximately 1400 hours of hospital day therapy unit chair time, creating greater capacity in the health care system for patients unable to self-administer their medication at home and a saving of approximately Aus $71,000 (US $47,229.20) for the hospital [13].

However, while interventions can work well in one context, scaling to other settings can be problematic. This may be due to various factors that can influence the uptake of an intervention, such as complex health systems, funding constraints, personal preferences or motivations, and workforce challenges [14]. Other MM teams across Australia have been unable to initiate or sustain an NEST-SP. Anecdotal reasons reported for this include inflexible systems, short expiry times on in-house compounded bortezomib, risk management considerations, beliefs about patient capability, and resourcing.

With multiple SC immunotherapies, including anti-CD38 monoclonal antibody and T cell–redirecting antibodies, including bi- and trispecific antibodies, in development for MM [15], there is considerable opportunity to expand NEST-SPs to benefit patients across Australia and worldwide. Studies to expand understanding of the barriers to and enablers of implementing complex health interventions such as new models of care that potentially offer patient- and system-level benefits are urgently needed.

### Using Implementation Science to Capture Broad-Ranging Complexities

Implementation science (IS) provides a systematic approach to understanding factors that influence the uptake of evidence-based interventions in practice [14]. Evidence-based IS frameworks offer a theory-informed approach to understanding and addressing factors that hamper effective implementation by exploring the barriers to and enablers of intervention uptake. By making barriers and enablers known, it is possible to develop new or apply evidence-informed interventions or strategies tailored to strengthening enablers and overcoming barriers [16].

This study will use the Consolidated Framework for Implementation Research (CFIR; described below) [17,18] to allow for the systematic exploration and description of barriers to and enablers of implementing NEST-SPs for patients with MM. Identified barriers will be mapped to the CFIR Expert Recommendations for Implementing Change (ERIC) matching tool [19]. The data generated will be used to inform future development of a national implementation road map to facilitate nationwide scaling of NEST-SPs.

The study objectives are as follows:

1. To develop an understanding of barriers to and enablers of implementing nurse-enabled SC immunotherapy self-administration programs for patients with MM.
2. To identify potential strategies to address these barriers and enhance enablers of program implementation in other contexts.
3. To generate data to inform future development of a national road map for implementation of an NEST-SP.

## Methods

### Qualitative Approach

We will undertake a qualitative descriptive study informed by a pragmatic research approach [20]. Pragmatism as a research paradigm is based on the assumption that researchers choose the method or design that best addresses the needs of the problem being researched and that knowledge is based on experience [21]. As the intent of this study is to learn from the experiences of those who have attempted to implement or have received care through NEST-SPs, a pragmatic paradigm aligns well [22]. A qualitative approach was chosen to enable in-depth exploration of a topic about which little is known [23] and enable purposeful exploration of participant experiences through consideration of CFIR constructs to generate a novel and comprehensive understanding of barriers to and enablers of the implementation and sustainability of NEST-SPs.

In keeping with best practice principles of undertaking qualitative research [24], reflexivity will be a key consideration throughout the study to address transparency and rigor [25]. We acknowledge that the study team comprises clinicians and researchers with extensive experience in myeloma and nurse-enabled interventions and a shared belief in the potential benefits of NEST-SPs, which may influence data collection and interpretation. Strategies to mitigate this include self-interview and structured team reflexive discussion [25]. The manuscript complies with the Standards for Reporting Qualitative Research checklist [26] relevant at the study protocol stage.

### Study Population

Patients with MM, carers (to include family members or friends), multidisciplinary health care professionals (HCPs) who are experts in the management of MM, policymakers, and industry partners from across Australia were recruited. This breadth of perspectives is required to reflect the complexity of factors that need to be considered across all domains of the CFIR to establish the barriers to and enablers of the program.

Patients had a diagnosis of MM and experience receiving SC therapy either through an NEST-SP or as administered by a health professional. Carers will have experience supporting a patient through a diagnosis of MM and SC therapy.

HCPs had experience caring for patients with MM and the process of administering SC therapies through either an NEST-SP or a hospital chemotherapy day unit.

The policymakers were representatives from government and regulatory agencies, senior hospital administration, and the pharmaceutical industry to reflect the perspective of NEST-SP development that relates to factors beyond the chemotherapy day unit.

### Sample Selection and Recruitment

A purposive sampling [27] approach was used to ensure the recruitment of individuals with relevant experience in and knowledgeable about SC therapy delivery in MM. Stakeholder mapping was used to create a list of potential HCP and policymaker participants from HB’s network and checked for completeness by the study team to ensure inclusion of all required stakeholders. Recruitment of patients and carers was advertised through Myeloma Australia, a national nongovernmental organization.

Informed by the work of Hennink et al [28], and aligned with the choice of applying a deductive framework analysis approach to the data to be generated [29,30], we aimed to undertake 6 distinct focus groups, each one exploring the perspectives of diverse stakeholder participants (Textbox 1). Evidence from rigorous assessment of the number of focus groups required to generate credible deductive codes indicates that 1 focus group yields over 60% of deductive, high-prevalence codes per stakeholder group, with considerable decline in the number of codes generated in each focus group conducted after this [28]. For our study, 1 focus group per stakeholder grouping (stratum) to a maximum of 6 focus groups was determined to be adequate to achieve code saturation (capturing most and the most prevalent codes relevant to the CFIR domains being explored) [[31]]. We aimed to recruit a prespecified minimum and maximum number of participants to each of the 6 focus groups—detailed in Textbox 1. These numbers are informed by consideration of the study purpose (to identify barriers and enablers), generation of concrete codes (via a framework analysis informed by the CFIR), and stratification by stratum or group characteristics (Textbox 1) [28]. Although we recognize that interviews and focus groups generate different types of data and may impact how saturation is achieved [29], we offered the opportunity to take part in an interview for anyone who wishes to contribute but, because of work commitments or health concerns, is unable to attend a scheduled focus group.

Textbox 1. **Allocation of study participants into focus groups.**

**Focus group 1**

- Patients and carers with experience of a nurse-enabled subcutaneous therapy self-administration program (NEST-SP)
- Number of participants: minimum of 4 and maximum of 6
- Patients and carers (stratum: consumers)

**Focus group 2**

- Patients and carers without experience of an NEST-SP
- Number of participants: minimum of 4 and maximum of 6
- Patients and carers (stratum: consumers)

**Focus group 3**

- Health care professionals (HCPs) with experience facilitating an NEST-SP
- Number of participants: minimum of 5 and maximum of 8
- Hematologists, oncologists, nurses, pharmacists, and general practitioners (GPs; stratum: health professionals)

**Focus group 4**

- HCPs without experience facilitating an NEST-SP
- Number of participants: minimum of 5 and maximum of 8
- Hematologists, oncologists, nurses, pharmacists, and GPs (stratum: health professionals)

**Focus group 5**

- HCPs from a private health service
- Number of participants: minimum of 5 and maximum of 8
- Hematologists, oncologists, nurses, pharmacists, and GPs (stratum: health professionals)

**Focus group 6**

- Policymakers
- Number of participants: minimum of 4 and maximum of 6
- Senior hospital administrators and representatives of Cancer Australia, the Department of Health, Disability, and Aging, and the pharmaceutical industry (stratum: industry, administrators, and policy)

Email invitations to participate were sent with a participant information letter. The invitation included an overview of the study and an outline of participation requirements. There was one participant information letter for patients and carers and another for HCPs, industry partners, and government officials. The participant information letter included contact details should a potential participant wish to ask any questions before deciding to take part. The email also included a link to a secure online form for collecting informed consent and demographic data and offered the option to receive a copy of the results at the end of the study.

### Data Collection

To gather comprehensive perspectives, we conducted focus groups and, if necessary to achieve minimum numbers for each stakeholder group, semistructured interviews to address the study objectives.

Following ethics approval and consent, participants were allocated to a focus group according to expertise and experience facilitating or participating in an NEST-SP. Participants who were unable to attend a focus group were offered a semistructured interview. An explanation of how participants were allocated to focus groups can be found in Textbox 1.

Focus groups (or interviews) were conducted using study-specific schedules informed by the CFIR and developed by the project team. A series of schedules appropriate to each stakeholder group was developed and checked for clarity and completeness of issues to be explored by the project team ahead of data collection. An example of a focus group schedule can be found in Table 1.

**Table 1.** Example of a focus group schedule based on the Consolidated Framework for Implementation Research domains and constructs (focus group 1: patients and carers with experience of a nurse-enabled subcutaneous therapy self-administration program).

Domain and construct	Focus group questions
Innovation domain	
Innovation adaptability construct	“How does the program work at your hospital?”
Innovation design construct	“Is there anything you can think of that would make the experience better?”
Innovation evidence construct	“What kind of information or evidence did you use or would have liked to have when deciding whether to take part in the program?”
Outer setting domain	
Critical incidents construct	“For those receiving bortezomib during the COVID-19 pandemic, was the self-administration program impacted at all? If yes, in what way?”
Inner setting domain	
Structural characteristics construct	“Have you experienced any difficulties in participating in the program?”
Physical infrastructure construct	“Can you think of anything that would make the program easier for you to access or adhere to?”
Individuals domain	
Implementation facilitators construct	“Which health professionals from the hospital do you have contact with as a participant in the program?”
Innovation recipient construct	“How confident were you that would be able to self-administer your bortezomib injections? Why/why not?”
Implementation process domain	
Sustainability construct	“Is there anything about the program that you think would make people not want to take part?”

### IS Framework

The CFIR [17] was chosen to inform key aspects of the study design, including selection of study participants and data collection, analysis, and reporting [17].

The CFIR comprises 5 domains: innovation, outer setting, inner setting, individuals, and the implementation process. Within each domain sit several constructs that describe different aspects of the implementation process as they relate to each domain [17].

Table 2 outlines the ways in which the CFIR domains relate to and guide the study design.

**Table 2.** Consolidated Framework for Implementation Research (CFIR) domains as they relate to the study design.

CFIR domain	Study-specific description
Innovation	All elements related to the innovation being implemented (the NEST-SP^a^; eg, source, evidence base, trialability, adaptability, complexity, design, and cost)
Outer setting	All factors influencing facilitation of the program that sit outside the inner setting (eg, local attitudes and conditions, policies, financing, and external pressures to implement an NEST-SP)
Inner setting	All factors related to the direct implementation and facilitation of the NEST-SP (eg, drug prescribing, drug dispensing, drug delivery, patient and carer education, and patient monitoring)
Individuals	All key stakeholders—including patients, carers, physicians, nurses, pharmacists, senior hospital administrators, pharmaceutical industry representatives, and government officials
Implementation process	All factors related to the process of implementation of the NEST-SP (eg, needs assessment, planning, engagement, adaptation, and evaluation)

a NEST-SP: nurse-enabled subcutaneous therapy self-administration program.

Focus groups and interviews were conducted virtually using Zoom (Zoom Video Communications) and were audio and video recorded to allow for transcribing. Transcriptions will be checked for accuracy by a research assistant (RA), and in each transcript, participants will be deidentified and assigned a unique identifier.

Each focus group or interview ran for approximately 60 to 90 minutes and was facilitated by 2 members of the study team (HB and an RA). Consent was reconfirmed with participants verbally before focus group or interview commencement, and each focus group participant was asked to maintain confidentiality regarding any information shared during the discussion.

### Data Analysis

All data will be transcribed verbatim, deidentified, and entered into NVivo (version 14; Lumivero; an online qualitative data management software platform) [30] to organize and support analysis and visualization.

The framework method approach will be used to organize and analyze the data. This 7-step approach includes transcription, familiarization, coding, development and application of a working analytical framework, and charting and interpreting the data [32]. Framework analysis is a qualitative technique initially developed for policy and health research in the United Kingdom [33]. It is more prescriptive than other research methodologies, providing a 7-step approach to systematically analyze data by participants and themes using a data matrix. In the matrix, rows represent stakeholder groups, and columns represent themes, enabling researchers to compare data across participants and themes as data analysis progresses. A deductive approach will be used to code transcripts, organize data, and assign data according to CFIR domains and constructs as part of the data matrix [32].

Study-specific definitions for CFIR constructs developed by the project team members will be used to guide application of the framework method for analysis of the focus group and interview data [32]. An example of how this technique will be applied in this study is provided in Multimedia Appendix 1.

Data will be analyzed within and across each of the participant stakeholder groups to explore similarities and differences regarding barriers and enablers. Two members of the project team (HB and an RA) will analyze and code the first 2 transcripts to assess intercoder reliability. Any differences in opinion will be resolved by inviting another member of the project team to independently code the data extract. The remaining transcripts will be coded by one member of the project team (HB).

Interview transcripts will be coded according to the domains of the CFIR. This analysis will take place in 2 stages: a deductive approach to sort participant data against the CFIR domains and an inductive approach to populate the domains with content themes as evident in the qualitative data. Barriers and enablers will be compared within and across groups and stakeholders to identify similarities and differences. All barriers will be mapped to the CFIR ERIC matching tool at this stage. Consideration of prioritization of ERIC strategy selection will take place in the next phase of this work (to be detailed in a future protocol) [19]. While we recognize that a dominant deductive approach to analysis may limit emergent themes relevant to the CFIR domains, the next stage of this work will provide the opportunity for a different group of key stakeholders to add to and further expand the understanding of barriers and enablers obtained from participants in this study, further strengthening the credibility of the data generated.

At the completion of this analysis, the data will be used to populate and develop the implementation road map. An evidence-informed framework such as the National Health and Medical Research Council’s guide to the development, implementation, and evaluation of clinical practice guidelines [34] will be used to inform this phase of the work.

### Ethical Considerations

Ethics approval was granted by the Peter MacCallum Cancer Centre Human Research Ethics Committee (approval HREC/107517/PMCC). Informed consent is mandatory for all participants via an online form. Focus group and interview transcripts will be deidentified to maintain participant anonymity and kept in password-protected folders only accessible to study staff.

As outlined in the participant information letter, patient and carer participants were informed that, if they find any questions upsetting or they do not wish to answer a question during the focus group or interview, they can wait for the next question or choose to leave the focus group or interview. If a participant decided to leave a focus group, a member of the study team made contact at the conclusion of the focus group to offer support as needed. Contact details for the consumer liaison officer (patient and carer participants) and local ethics committee representative (health professional, administration, policy, and industry participants) are included in the participant information letter. If a patient or carer became distressed because of disease-related issues, they were referred, with their permission, to Myeloma Australia’s telephone support line service. If the withdrawal of a participant resulted in a focus group falling below the minimum group composition requirements (Textbox 1), we saw to recruit an appropriate replacement.

At the beginning of each focus group, participants were reminded that any personal information shared during the discussion must be kept confidential. They were assured that there is no right or wrong answer or perspective and that all responses are valuable. If any sensitive data were disclosed intentionally or unintentionally, the group was reminded of the confidential nature of the discussion. If any concerns arose as a result of disclosure (eg, patient safety or issues relevant to practice), these were discussed among the study team, and a documented plan to respond was developed and enacted.

Data will be stored in accordance with National Health and Medical Research Council guidelines for conduct of research for the duration of the project and 5 years after completion of the final publication in accordance with best practice research guidelines.

Participants were not incentivized to take part in this study.

## Results

This study was funded in March 2024 and approved by the institution ethics committee in May 2024. Data collection was conducted between June 2024 and November 2024. A total of 76.2% (32/42) of the approached participants were recruited. There were 6 focus groups and 10 semistructured interviews carried out. Participant numbers per focus group ranged between 3 and 7. Data analysis is nearing completion, with results expected to be published in February 2026.

## Discussion

Insights from the development, implementation, and facilitation of our NEST-SP demonstrate that NEST-SPs require multidisciplinary collaboration and key stakeholder engagement that vary across health care settings and teams.

To our knowledge, this study will be the first of its kind to identify and compare barriers to and enablers of implementing NEST-SPs for patients with MM. Applying the CFIR to guide the study provides an evidence-informed structure to understand how discrete and intersecting factors influence program implementation [35].

Evidence generated from this study will be used to inform early development of an implementation road map to support national implementation of NEST-SPs for people affected by MM. The road map will undergo further development by stakeholders from diverse settings in subsequent studies building on this research.

The implementation of an NEST-SP has the potential to improve patient experience and quality of life by allowing people to spend less time in hospital, reducing the time [36] and financial toxicity [11] of unnecessary travel and addressing a commonly reported unmet need among people with cancer—“to be able to take care of myself” [7,8]. Furthermore, evidence indicates that nurse-enabled care may enhance patient self-management capacity [37] for patients with cancer and other chronic conditions [38]. Importantly, approaches to care that enable self-management may address inequities in care experienced by patients living in regional, rural, and remote locations where time and cost toxicity impact access to and acceptability of care, potentially impacting treatment decision-making [39] and disease-specific outcomes.

As the availability of SC therapies for other cancers and chronic diseases grows, the implications of this work have the potential to extend beyond MM. Importantly, in the next study building on this work, participants from regional cancer centers in Australia will be recruited to consider, add to, and further strengthen the findings of this research. Their perspectives will help inform equitable application and opportunity for implementation of this initiative across diverse health settings and resources, ensuring its applicability and utility for cohorts of patients with cancer in rural and remote areas known to experience poorer cancer outcomes than those of their metropolitan counterparts [40,41].

This study will improve our understanding of the barriers and enablers experienced across Australian health services seeking to implement NEST-SPs. The data generated will help inform future approaches to effective implementation of NEST-SPs. The application of the CFIR framework is novel for this work, providing a structured, evidence-based approach to exploring barriers to and enablers of innovations in health care delivery.

## Supplementary material

10.2196/85053 Multimedia Appendix 1 Example of a data coding matrix.

## Abbreviations

CFIR: Consolidated Framework for Implementation Research
ERIC: Expert Recommendations for Implementing Change
HCP: health care professional
IS: implementation science
MM: multiple myeloma
NEST-SP: nurse-enabled subcutaneous therapy self-administration program
RA: research assistant
SC: subcutaneous
