# Supplementary material for: Establishing Barriers to and Enablers of Nurse-Enabled Subcutaneous Therapy Self-Administration Programs for Patients With Myeloma: Protocol for a Qualitative Descriptive Study
Source: JMIR Res Protoc. 2025 Dec 23;14:e85053. doi: 10.2196/85053 (PMC12724064; doi:10.2196/85053)
Supplement: Multimedia Appendix 1 [file resprot-v14-e85053-s001.docx]

Multimedia Appendix 1. Example of data coding matrix

| Domain | Outer setting | | |
| --- | --- | --- | --- |
| Construct | Local attitudes | Barrier | Enabler |
| Stakeholder group | Patients and Carers with experience of a NEST-SP |  |  |
|  | Patients and carers without experience of a NEST-SP |  |  |
|  | HCPs with experience of a NEST-SP |  |  |
|  | HCPs without experience of a NEST-SP |  |  |
|  | HCPs from a private health service |  |  |
|  | Policy makers |  |  |
